# Rapid Syphilis Tests as Catalysts for Health Systems Strengthening: A Case Study from Peru

**DOI:** 10.1371/journal.pone.0066905

**Published:** 2013-06-26

**Authors:** Patricia J. García, César P. Cárcamo, Marina Chiappe, Maria Valderrama, Sayda La Rosa, King K. Holmes, David C. W. Mabey, Rosanna W. Peeling

**Affiliations:** 1 Epidemiology, STD, and HIV Unit, School of Public Health and Administration Universidad Peruana Cayetano Heredia, Lima, Peru; 2 Department of Global Health, University of Washington, Seattle, Washington, United States of America; 3 London School of Hygiene and Tropical Medicine, London, United Kingdom; Fundacion Huesped, Argentina

## Abstract

**Objectives:**

Untreated maternal syphilis leads to adverse pregnancy outcomes. The use of point of care tests (POCT) offers an opportunity to improve screening coverage for syphilis and other aspects of health systems. Our objective is to present the experience of the introduction of POCT for syphilis in Peru and describe how new technology can catalyze health system strengthening.

**Methods:**

The study was implemented from September 2009–November 2010 to assess the feasibility of the use of a POCT for syphilis for screening pregnant women in Lima, Peru. Outcomes measured included access to syphilis screening, treatment coverage, partner treatment, effect on patient flow and service efficiency, acceptability among providers and patients, and sustainability.

**Results:**

Before the introduction of POCT, a pregnant woman needed 6 visits to the health center in 27 days before she received her syphilis result. We trained 604 health providers and implemented the POCT for syphilis as the “two for one strategy”, offering with one finger stick both syphilis and HIV testing. Implementation of the POCT resulted in testing and treatment on the first visit. Screening and treatment coverages for syphilis improved significantly compared with the previous year. Implementation of POCT has been scaled up nationally since the study ended, and coverages for screening, treatment and partner treatment have remained over 92%.

**Conclusions:**

Implementation of POCT for syphilis proved feasible and acceptable, and led to improvement in several aspects of health services. For the process to be effective we highlight the importance of: (1) engaging the authorities; (2) dissipating tensions between providers and identifying champions; (3) training according to the needs; (4) providing monitoring, supervision, support and recognition; (5) sharing results and discussing actions together; (6) consulting and obtaining feedback from users; and (7) integrating with other services such as with rapid HIV testing.

## Introduction

Mother-to-child transmission of syphilis continues to be a problem, with more than 2 million pregnancies each year in women with syphilis [1]. In at least 69% of them, adverse pregnancy outcomes are estimated to happen, including congenital syphilis, miscarriage, stillbirth, low birth-weight, and preterm labor. Treatment with a single dose of penicillin before 28 weeks gestation prevents these adverse outcomes [2]–[4].

Most countries have existing policies for universal syphilis screening during pregnancy, but implementation of such policies is weak, especially in developing countries, where congenital syphilis remains a major public health problem [5]. Numerous barriers have been identified which limit the effective screening and treatment of pregnant women: poor access to antenatal care (ANC), lack of tests at the facilities, cost of screening tests, and delays in providing results of the serological tests which often means losing the opportunity of treatment. The point-of–care tests (POCT) for syphilis available now are treponemal tests, which detect antibodies to treponemal antigens. Although treponemal tests cannot be used to distinguish between active and past treated infection, these tests do not require refrigeration, are affordable, easy to perform, and can give results in 15–20 minutes, allowing same-day testing and treatment [6]. In six low-middle income countries, including Peru, introduction of POCT into health services to improve syphilis screening proved feasible, and resulted in increased proportions of pregnant women being screened and treated [7]. This paper presents the experience of the introduction of POCT for syphilis in Peru to illustrate this technology as catalyst for improving health delivery and health systems. Health systems strengthening, as any array of initiatives and strategies leading to improvements in access, coverage, quality or efficiency [8], has been identified as one of the critical steps towards the global elimination of congenital syphilis [5]; [9].

## Materials and Methods

### Ethical Statement

Approvals from the Ethics Review Committees from the World Health Organization, Universidad Peruana Cayetano Heredia (Lima, Peru), University of Washington (Seattle, WA, USA), Callao Directorate of Health, and INMP were obtained. All participants signed informed consents.

During 2009–2010, as part of a multi-country project [7], we implemented an operations research study to assess the feasibility of using a POCT to screen pregnant women in Lima, Peru, for syphilis.

The Peruvian study was named, “CISNE” (which in Spanish means SWAN and stands for the Spanish acronym for *Immediate Cure for Neonatal Syphilis*). During a baseline focus group, women were asked about options for possible project names and suggested the name CISNE. They explained that, when a woman is infected with syphilis, the associated stigmatization makes her feel like an “ugly duckling.” Once she is diagnosed and receives treatment, however, she is transformed into a beautiful swan.

A POCT for syphilis (SD Bioline 3.0®, Standard Diagnostics Inc. Korea) was introduced in the public sector antenatal and reproductive health services (including antenatal, delivery, postpartum, and miscarriage services). Intervention sites included the largest maternity hospital in Peru, the National Maternal Perinatal Institute (INMP) in Lima, and a network of 15 semi-urban health centers and one hospital in Ventanilla-Callao, a resource-poor district north of Lima.

The study lasted 14 months and was divided into two phases. The preparation phase, from September to December 2009, included ethical approvals, coordination meetings with authorities and health professionals, baseline collection of information, development of standard operating procedures (SOP), and development of training materials. The implementation and follow-up phase started January 2010 and ended November 2010. Overall success of implementation was measured by outcomes including syphilis screening and treatment coverage, partner treatment, effect on patient flow and service efficiency, acceptability of POCT among providers and patients, and sustainability.

### Meetings with Authorities

Prior to and during the initial phase of implementation, a total of 37 meetings were held with local and national authorities. These meetings provided opportunities to involve them in the study design and to play an active part in the roll-out of POCT screening over the course of implementation.

### Working Meetings with Other Key Health Workers

To coordinate logistical details of the upcoming implementation and the follow up of the achievements, 215 working meetings were also held during the same period with local personnel who would be involved in POCT implementation. These included the coordinators of health centers and programs for reproductive health and STDs, heads of the local laboratories, and heads of the pharmacy programs, logistics offices, and midwives.

### Baseline Data

We collected national and local data from 2010 on screening coverage with RPR for pregnant women, and treatment coverage for syphilis-seroreactive women. We also collected information on health care providers' knowledge about syphilis and explored perceptions related to the introduction of POCT. The information gathered was used to determine training needs, how tests would be integrated into the existing flow of care, what category of health professionals would perform the test, and the counselling women would receive in conjunction with the test.

The CISNE team completed a mapping process in the health network of Ventanilla, to understand the current flow of care at health establishments, and how POCTs could be effectively integrated into the existing algorithms of care. The mapping process included: (1) interviews with health care providers; (2) simulated patients who recorded each of the steps to complete an ANC visit; and (3) real patients interviewed and then invited to participate, documenting the process of seeking ANC including waiting times and barriers encountered with each step of the process.

### Training materials and training

Materials were prepared for training, implementation, and dissemination of information. Educational materials included brochures, waiting room posters, flip charts, and a short video on the importance of syphilis screening to be shown (and now still being shown) in the waiting rooms of intervention health establishments. All materials are available through our website www.proyectocisne.org.

Regular ANC is mainly performed at the peripheral centers by professional midwives, so they were the main focus for training. A total of 604 midwives and nurses and laboratory workers were trained during the project. Training included clinical information on syphilis, treatment regimens, performing and interpreting the syphilis POCT, biosafety, and appropriate test reagent storage. Since these POCT for syphilis are read by eye and are subjective, we added as a regular component to the training workshops an assessment visual acuity for reading and color vision.

### Implementation

In Ventanilla, the concept of **“two tests one stick, or two for one”** was introduced to integrate syphilis and HIV screening with POCTs.

At all intervention sites, the **Same Day Testing and Treatment (STAT)** **strategy** was applied with the objective that any pregnant woman with a positive POCT syphilis test result would receive the first dose of treatment on the day she is tested.

### Data collection during the intervention

We performed complementary evaluations comparing cost-effectiveness of POCT against the Rapid Plasma Reagin (RPR) test; a performance analysis of POCT; and a post-implementation study of the acceptability of POCT among providers and patients. At the ANC services, midwives had a registration form provided by the Ministry of Health to record HIV and Syphilis testing and treatment in pregnant women. During the study we monitored the completeness and accuracy of the data collected, and added the information of partner treatment. After the study we continue using those forms to capture information.

## Results

### Baseline Data

**Figure 1:**
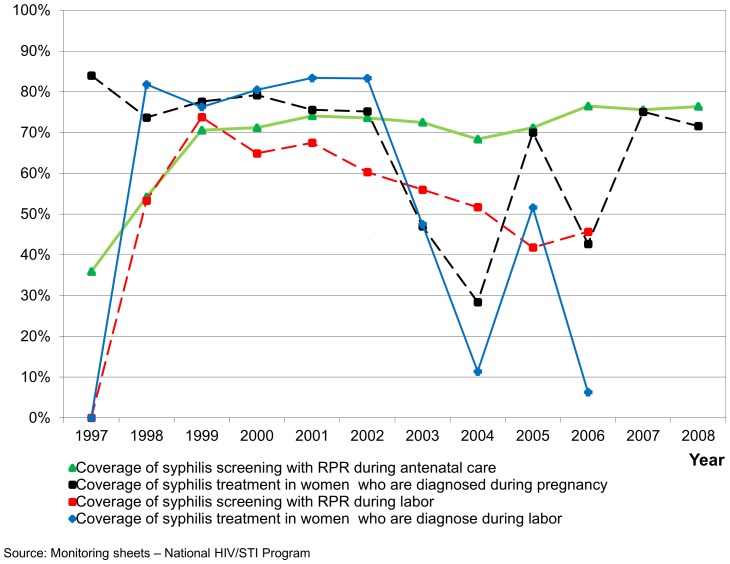
Syphilis screening and treatment in pregnant women, antenatal care and labor. **Peru (1997–2008).** Nationally the coverage for syphilis screening in pregnant women in antenatal care has been 75% since 2000, but treatment coverage for syphilis seropositive pregnant women fallen intermittently, from over 80% in 2000, to as low as 30% in 2004. Screening for syphilis for women during labor has been low as well as the treatment of women found seropositive in this situation.

At the national level screening coverage for syphilis in pregnant women, according to the reports from the National HIV/STI Program, has been 75% since 2000, but treatment coverage for syphilis seropositive women in ANC had fallen intermittently, from over 80% in 2000, to as low as 30% in 2004 (Figure 1). Prevalences of maternal syphilis varied. According to this same source, the mean maternal syphilis seroprevalence, by RPR test, was 1.3% in ANC between1997–2004. A higher prevalence was reported for women screened at labor (from 1.5% to 3.1%). Screening at labor is policy only when pregnant women have not attended ANC. Treatment coverage for women who were seropositive during labor fell dramatically from over 80% in 2002 to under 10% in 2006.

#### Poor knowledge about syphilis and tensions among health providers

During baseline surveys, providers demonstrated poor knowledge and little recognition of syphilis as a problem. Some health workers had worked with rapid tests for HIV. Their initial experience with rapid tests for HIV was reported as very bad, due to the high rates of false positives, which make them refer to rapid tests as “unreliable.” Several providers therefore shared their fear and distrust of any diagnostic test called rapid, saying “good things take time.” These health workers were hesitant about the introduction of a POCT syphilis test as it was seen similar to the “rapid HIV test.”

Additionally, laboratory workers felt the test could compromise their “authority,” while clinicians were concerned that a POCT for syphilis would increase their workload.

In order to dissipate fears, we defined different responsibilities for each provider in the implementation pathway, i.e., midwives screening in ANC and labor; nurses screening at miscarriage services; physicians prescribing the treatment; laboratory workers in charge of the quality control system; and pharmacists (within health centers) to ensure that the test and the penicillin were always available.

#### Mapping of the antenatal care services (ANC)

According to Peruvian National Guidelines for Care of Sexual and Reproductive Health [10], the first ANC visit should include not only physical examination, but also a set of basic tests: blood typing, haemoglobin or hematocrit, glucose, syphilis test, HIV test, and a urine test.

Through the mapping process we found that to complete an ANC visit, a pregnant woman had to go to a health center a total of 6 times during a total of 27 days. HIV rapid testing was implemented in the country several years ago, but the test was performed with venous blood and at a central laboratory. In a case of a positive syphilis test (which was commonly an RPR test) there was a delay of almost a month between screening and treatment if penicillin was available, which we found was not always the case.

These findings were presented to the district MOH officials, and helped lay the groundwork for stakeholder support throughout the implementation process.

### Training

We found that 269 of 604 (44.5%) health providers participating in the training had difficulties with near vision which did not allow them to recognize the line in the POCT. Two (0.3%) were color blind, and did not recognize the red line. For the first group we were able, in coordination with the local authorities, to recommend and prescribe visual aids (reading glasses) based on this evaluation and the health provider's visual needs. We found it most helpful, when implementing this POCT, to include the visual evaluation, which improved the performance of the providers reading the tests, once the visual problem was corrected.

After implementation began, the research team continued to hold coordination and training meetings as appropriate, in order to strengthen skills and dispel doubts related to the use of the syphilis POCT. Throughout the study period, it became increasingly clear that the physicians (who are not necessarily involved in the ANC but determined treatment regimes for patients with syphilis) also needed to be sensitized about POCT, despite the fact that they were not directly involved in the performance of tests. We held a series of five training sessions for physicians in the INMP and Ventanilla.

### Implementation results

**Figure 2:**
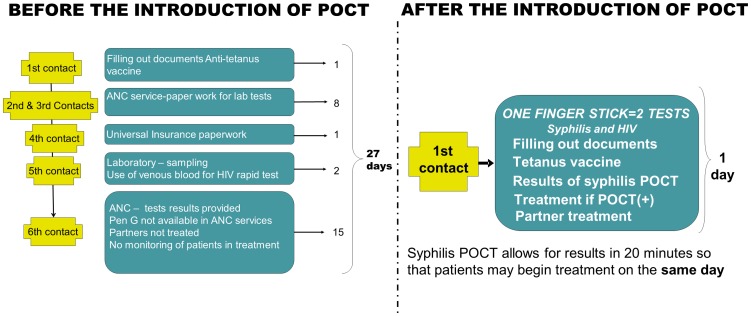
First antenatal care visit: Changes before and after the implementation of POCT for syphilis (Ventanilla-Callao). Before the implementation of the POCT for syphilis, women needed 27 days to have the HIV and Syphilis tests results as part of the first antenatal care visit (ANC) visit. After the implementation, the testing and results are done on the same visit.

The implementation of the POCT in all locations resulted in a change of the patient flow for ANC, and the offer of the testing and treatment for HIV and syphilis on the first visit. We documented a decrease from six visits to one (Figure 2).

### Screening Coverage and Treatment

From a total of 18,105 women, 17,155 were screened with syphilis POCT in Ventanilla and the INMP. A total of 154 (0.9%) women had a positive POCT for syphilis. None of the seropositive women had lesions or symptoms of active infection. When asked, 48/154 (31.2%) reported a previous history of syphilis and none recall previous treatment. Screening coverage was 94.8% and treatment coverage with at least one dose of penicillin was 91.6%. Eighty percent received at least two doses of penicillin.

As can be seen in Table 1, screening and treatment coverage were higher at ANC services, lowest at miscarriage services.

**Table 1:** Screening and treatment coverage with POCT for syphilis at INMP and in Ventanilla-Callao (Jan-Nov 2010).

Services	N Pregnant women	Screened pregnant women	Screening coverage	POCT+ (%)	Treatment coverage
Antenatal care	8882	8728	98.3%	83 (0.95)	94.9%
Miscarriage services	3687	3323	90.1%	28 (0.84)	88.9%
Delivery and emergency services	5536	5104	92.2%	43 (0.84)	86.5%
**Total**	**18105**	**17155**	**94.8%**	**154 (0.90)**	91.6%

**Figure 3:**
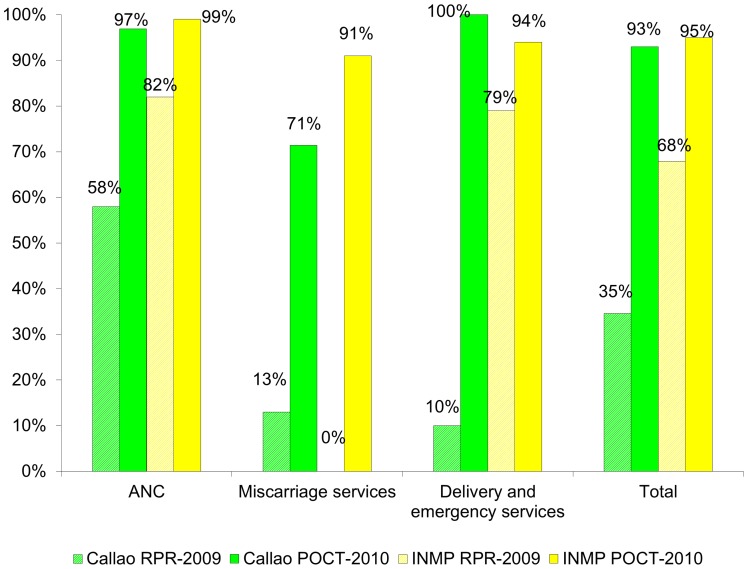
Comparative screening coverage: Ventanilla-Callao and INMP 2009 by RPR and 2010 by syphilis POCT. Syphilis screening coverage improved at the peripheral network of health centers in Ventanilla from 35% to 93% and at the INMP from 68% to 95% from 2009 to 2010.

Screening coverage improved at the peripheral network of health centers in Ventanilla from 35% to 93% (p<0.001) and at the INMP from 68% to 95% (p<0.001) from 2009 to 2010 (Figure 3). Treatment coverage for women increased from 40% to 100% (p<0.05) from February to the end of the study. Most of the cases of women not treated had to do with the reluctance of the physicians to accept the POCT results, especially at the beginning of the study. Partner treatment was not recorded before the study, but was an average of 52% during the implementation, with a coverage of 87% in the last month recorded.

### Dual HIV-Syphilis Testing (The “two for one” strategy)

The syphilis POCT was only integrated into the well-established rapid testing program for HIV only in the Ventanilla Health Network as a model. At the first ANC visit, women were screened for both tests with one fingerstick. Integrating the two tests improved uptake of the syphilis POCT and simplified processes. A total of 4497 women were screened with the HIV rapid test and the POCT for syphilis during the study period. Thirteen (0.3%) women tested HIV positive and 43(1%) positive for syphilis. No cases of syphilis-HIV co-infection were found.

During the implementation period we documented difficulties regarding stockouts and the use of multiple types of rapid HIV test by the health centers. Due to the fact that the tests are bought locally and in small quantities, stockouts are common, the “format” of the test varies, no quality control is available, and each time a new test is introduced there is no training for the health workers. Tests vary in the time for incubation, and the number drops of buffer added to the wells. We documented also the poor quality of the tests, with 15% of invalid in some brands of rapid HIV test. This situation was shared with the authorities, and the lessons learned were used to introduce a system of central procurement for the syphilis POCT and improve the available system for HIV.

### Transfer and Scale-Up of syphilis POCT screening, what has happened?

During the study the testing was done by Ministry of Health (MOH) personnel, with supervision from the CISNE team. Three months prior to the conclusion of the study, a plan was put in place for the gradual transfer of supervision from the CISNE team to the MOH administrators and health establishments. The plan outlined how CISNE supervision slowly decreased over time, and responsibilities were transferred to the public sector and how the scale up will be done.

Due to their involvement in the planning and follow up of the study, the local directorate authorities and authorities from the MOH witnessed the tangible improvements made possible through the POCT, and became vocal proponents for the inclusion of the syphilis POCT in guidelines governing prevention of congenital syphilis.

The first policy change that occurred as a result of the research was the revision of the INMP's institutional guidelines, this being the main national training institution on issues of maternal health. The second policy change, and the more important, is the one we have promoted at National level. After several meetings with the MOH authorities and members of the Reproductive Health and STI/HIV Programs, the syphilis POCT was introduced in the new “Guidelines for the Prevention of Mother-to-Child Transmission of HIV and Syphilis.” The Minister of Health launched in February 2011 the campaign “Yes to life, No to syphilis.” We collaborated with the MOH on the training at the national level, identifying the providers with the best performance, and “champions” within the project, and training them to become “trainers of trainers.” The syphilis POCT is now implemented in all centers in Peru, for screening in ANC, labor and miscarriage services.

CISNE's successful involvement of key stakeholders throughout the process of implementation resulted in immediate change in policy and practice to include syphilis POCT in national syphilis screening algorithms. We continued following the development of the use of the POCT for screening in the areas where we started. Coverages for screening, treatment and partner treatment have remained over 92%.

We have been able to catalyze the interactions between cooperation agencies such as PAHO and UNICEF and the MOH, and with their support the MOH of Peru bought the first 500,000 tests for 2011 and national distribution of the tests was performed. For 2012 the supplies for syphilis POCT have been bought and distributed nationally.

We collaborated with the London School of Hygiene and Tropical Medicine in the development and adaptation of a “Rapid Syphilis Test Toolkit” which is a document including tools for the introduction of rapid syphilis tests into country programs [11]. A version in Spanish is also available through the CISNE web site (www.proyectocisne.org). Another resource available in Spanish is an online course on Maternal and Congenital Syphilis and POCT, which was elaborated with the INMP, and can be accessed at http://sicavirtual.vis.com.pe/campus/.

## Discussion

This implementation research project assessed the feasibility of introducing POCT for syphilis for antenatal screening and reproductive health services in Lima, Peru. It not only proved to be feasible, but the introduction improved several aspects of the health system. We were able to simplify processes, decrease the number of times a pregnant woman had to go to ANC, increase coverage of syphilis screening and treatment, improve information, and improve relations between providers by clarifying their roles within the system.

There are publications which refer to the implementation of syphilis POCT in developing countries and its successes [7]; [12]–[14], but our objective with this manuscript is to highlight how to make the implementation more successful and sustainable and how the introduction of the POCT could help to identify and improve health systems problems. There are several lessons learned that we would like to summarize here.

### 1. Engage the authorities and all stakeholders

Prior to and during to implementation we found it critical to meet with authorities from different levels and stakeholders (e.g. UNICEF, PAHO) to inform, discuss and make decisions about the project. Dealing with many institutions could be challenging, but bringing all stakeholders to the table issues like specific interests, discussing openly how best we could to work together and how to avoid duplications, helps. The time and effort spent on these meetings were well worth it and were largely responsible for the success of the program. In our case, important themes to discuss included syphilis and congenital syphilis prevalence, actual screening and treatment coverages, the need for improvement, treatment protocols and importance of partner treatment, performance of POCT compared with RPR, quality control issues, and general short and long-term goals for test implementation and data from the follow up and supervision. Throughout the study period, meetings were convened periodically to report progressive research results and answer questions arising. We involved all stakeholders wherever possible to cultivate their interest and increase long-term buy-in.

### 2. Dissipate tensions between providers, meet, discuss and identify champions

We found it extremely important to meet with all health care providers involved in the process of ANC and reproductive health prior to POCT implementation. Although the midwives were those who would be working directly with the test, we included all the levels of health professionals – nurses, physicians, lab technicians, and pharmacists – allowing us to dissipate tensions, overcome barriers, answer questions, clarify roles, and discover “champions” at all levels. Champions are those individuals who are proactive, interested in making things better, passionate in their job, capable of communicating well and to influencing their peers.

Those laboratory technicians who opposed the introduction of POCT changed their minds when they understood their key role in quality control; pharmacists helped to improved the logistics to assure penicillin availability at the clinic for treatment when needed, and nurses found their role in performing the POCT at the miscarriage services.

### 3. Train according to the needs and guide by baseline and follow up information

Quality training, based on the knowledge gaps and fears, proved essential to overcome barriers presented by health providers and administrators.

We highlighted the issues not known by the providers, that could motivate them to act, e.g.,“Syphilis is 20 times more frequent in Peru than HIV”; included practical sessions to learn how to perform fingersticks and how to read the test more accurately, sessions on the importance of quality control and a session directed to dispel myths and misbeliefs regarding maternal and congenital syphilis and the syphilis POCTs. One important issue that arose over the study period, however, was the refusal of some physicians to administer immediate treatment with a positive POCT because of doubts about test performance. Mainly they were those doctors who did not attend the training sessions. We had to incorporate “in situ” training (personal visits to those physicians to discuss the contents of the training session they missed) and created also the online course mentioned above. Furthermore, by widely diffusing information about syphilis POCT, it is likely that more providers will support implementation from the onset and the spread of misinformation will be kept to a minimum.

In addition to pre-implementation training, the CISNE team conducted periodic training sessions with health care providers to present results, reinforce key messages, and dispel doubts that had arisen along the way. These sessions were important to gain and maintain support from health providers. Furthermore, meeting periodically with providers created a feedback mechanism through which the study coordinators could glean information and make necessary changes.

### 4. Provide monitoring, supervision, support, and recognition

Especially during the first months of implementation, daily monitoring and supervision provided the support and accountability that providers needed to incorporate a new process into their already challenging workload. As the project evolved, the supervision frequency was lowered to once a week and later once a month.

Initially, providers resisted the added responsibility of syphilis POCT. Over time, however, we observed that they adapted to the process and came to recognize its merits. Daily “support” was indispensable to the change in attitudes over time. For this reason, as part of an implementation process of new technology (i.e., POCT), it is critical to include a team of well trained supervisors. We also included a system of recognition for their accomplishments, which included certificates of good performance given to the providers. They could receive a copper, bronze, silver, gold, or platinum certificate according their achievements, using different color papers, but very much appreciated! The award levels were successive and related to their performance during training or during supervisions, evaluations during quality assurance, training of other personnel and numbers of tests performed.

### 5. Share results and discuss actions together

We included in the monthly meetings that the midwives had as part of their regular activities a short section of “How is the POCT implementation evolving?” In five minutes, we presented the latest data on screening with the syphilis POCT and treatment in their establishments, from their records, and the findings of the supervision. Then we took ten minutes for discussion, to answer questions, and to recognize those doing a good job.

### 6. Consult and get feedback from the users

After some educational materials had already been printed with pictures of midwives performing POCT, other worker cadres (nurses, physicians) complained that the project had excluded them. We had to reprint the materials, this time including other providers in different activities related to the implementation. They helped in the design of these new booklets and also in the dissemination, and suggested new types of educational materials to develop such as posters and videos, which became very popular. This highlighted the need to consult health providers throughout the process of implementation, respect the complicated structure and processes present at each site, and take advantage of their experience.

### 7. Integrate with other services such as with HIV testing

Integration of syphilis and HIV screening with POCT is feasible and very well accepted by health workers and pregnant women in our study. The provision through the delivery of an integrated package increases synergy and efficiency since both aim at the same population of pregnant women. Time of the provider and the women can be saved doing the two tests at once; women only need one fingerstick; health services can save supplies (e.g. gloves, alcohol pads, lancets), the training for both tests can be done together and ANC and reproductive health services can be strengthened. If both tests are from the same company, procedures are likely similar, making training easier.

### Syphilis POCT implementation can strengthen health systems

According to the World Health Organization Report 2000, health systems consist of all the people and actions whose primary purpose is to improve health; the four key functions are: governance, financing, human and physical resources, and organization and management of service delivery [15]. Health system strengthening will lead to better health through improvements in one or more of the key functions, resulting in better access, coverage, quality, or efficiency.

## Conclusions

This study has shown that the use of the syphilis POCT is a feasible and acceptable maternal health intervention to prevent congenital syphilis in Peru. Rates of patient and partner screening and treatment during the study period show that implementation of POCT has the potential to increase timely screening and treatment coverage for syphilis for women. Through our research, policy makers and health care administrators were provided with applicable, current data with which to make evidence-based changes to existing policy. Furthermore, our study has demonstrated that POCT implementation can contribute to long-term improvements in service delivery, strengthening health systems and maternal and child health programs in Peru and potentially in other countries in the Latin American and Caribbean region.

## References

[1] McDermott J, Steketee R, Larsen S (1993) Syphilis-associated perinatal and infant mortality in rural Malawi. Bull World Health Organ 71: 773–780. 8313495 PMC2393540

[2] Hawkes S, Matin N, Broutet N, Low N (2011) Effectiveness of interventions to improve screening for syphilis in pregnancy: a systematic review and meta-analysis. Lancet Infect Dis 11: 684–691. 21683653 10.1016/S1473-3099(11)70104-9

[3] Watson-Jones D, Gumodoka B, Weiss H, Changalucha J, Todd J, et al (2002) Syphilis in pregnancy in Tanzania. II. The effectiveness of antenatal syphilis screening and single-dose benzathine penicillin treatment for the prevention of adverse pregnancy outcome. J Infect Dis. 186: 948–957. 10.1086/342951 12232835

[4] Saloojee H, Velaphi S, Goga Y, Afadapa N, Steen R, et al (2004) The prevention and management of congenital syphilis: an overview and recommendations. Bull World Health Organ. 82: 424–430. PMC2622853 15356934

[5] Kamb ML, Newman LM, Riley PL, Mark J, Hawkes SJ, et al (2010) A road map for the global elimination of congenital syphilis. Obstet Gynecol Int. 2010: 1–6. 10.1155/2010/312798 PMC2913802 20706693

[6] Herring A, Ballard R, Mabey D, Peeling RW (2006) WHO/TDR Sexually Transmitted Diseases Diagnostics Initiative: Evaluation of rapid diagnostic tests: syphilis. Nat Rev Microbiol. 4: S33–40. 10.1038/nrmicro1563 17366685

[7] Mabey DC, Sollis KA, Kelly HA, Benzaken AS, Bitarakwate E, et al (2012) Point-of-Care Tests to Strengthen Health Systems and Save Newborn Lives: The Case of Syphilis. PLoS Med 9: e1001233. 22719229 10.1371/journal.pmed.1001233 PMC3373627

[8] Health Systems 20/20 (2012) *The Health System Assessment Approach: A How-To Manual. Version 2.0.* http://www.healthsystemassessment.com/health-system-assessment-approach-a-how-to-manual. Accessed 2013 Feb 15.

[9] WHO (2007) *Every body's business: strengthening health systems to improve health outcomes WHO's frame work for action* Available: http://www.who.int/healthsystems/strategy/everybodys_business.pdf. Accessed 2013 Feb 15.

[10] Guías Nacionales de Atención Integral de Salud sexual y Reproductiva Lima, Perú. Ministerio de Salud (2004).Available: http://www.minsa.gob.pe/portada/publicaciondest.asp?dest_codigo=21.Accessed 2013 Feb 15.

[11] London School of Hygiene & Tropical Medicine (2011) Rapid Syphilis Test Toolkit: A Guide to Planning, Management and Implementation. Available: http://www.lshtm.ac.uk/itd/crd/research/rapidsyphilistoolkit/. Accessed 2013 Feb 15.

[12] Bronzan RN, Mwesigwa-Kayongo DC, Narkunas D, Schmid GP, Neilsen GA, et al (2007) On-site rapid antenatal syphilis screening with an immunochromatographic strip improves case detection and treatment in rural South African clinics. Sex Transm Dis. 34: S55–60. 10.1097/01.olq.0000245987.78067.0c 17139234

[13] García SG, Tinajeros F, Revollo R, Yam EA, Richmond K, et al (2007) Demonstrating public health at work: a demonstration project of congenital syphilis prevention efforts in Bolivia. Sex Transm Dis 34: S37–41. 17179776 10.1097/01.olq.0000251236.48770.35

[14] Benzaken AS, Sabidó M, Galban E, Pedroza V, Araújo AJ, et al (2011) Field performance of a rapid point-of-care diagnostic test for antenatal syphilis screening in the Amazon region, Brazil. Int J STD AIDS 22: 15–18. 21364061 10.1258/ijsa.2010.010145

[15] WHO (2000) The World Health Report 2000: Health Systems: Improving Performance. Available: http://www.who.int/whr/2000/en/. Accessed 2013 Feb 15.

